# The A4 study: *β*‐amyloid and cognition in 4432 cognitively unimpaired adults

**DOI:** 10.1002/acn3.51048

**Published:** 2020-04-21

**Authors:** Philip S. Insel, Michael C. Donohue, Reisa Sperling, Oskar Hansson, Niklas Mattsson‐Carlgren

**Affiliations:** ^1^ Clinical Memory Research Unit Faculty of Medicine Lund University Lund Sweden; ^2^ Department of Psychiatry University of California San Francisco California; ^3^ Alzheimer’s Therapeutic Research Institute Keck School of Medicine University of Southern California San Diego California; ^4^ Department of Neurology Harvard Aging Brain Study Massachusetts General Hospital Harvard Medical School Boston Massachusetts; ^5^ Department of Neurology Center for Alzheimer Research and Treatment Brigham and Women’s Hospital Harvard Medical School Boston Massachusetts; ^6^ Memory Clinic Skåne University Hospital Lund University Lund Sweden; ^7^ Department of Neurology Skåne University Hospital Lund University Lund Sweden; ^8^ Wallenberg Center for Molecular Medicine Lund University Lund Sweden

## Abstract

**Objective:**

To clarify the preclinical stage of Alzheimer’s disease by estimating when *β*‐amyloid accumulation first becomes associated with changes in cognition.

**Methods:**

Here we studied a large group (*N* = 4432) of cognitively unimpaired individuals who were screened for inclusion in the A4 trial (age 65–85) to assess the effect of subthreshold levels of *β*‐amyloid on cognition and to identify which cognitive domains first become affected.

**Results:**

*β*‐amyloid accumulation was linked to significant cognitive dysfunction in cognitively unimpaired participants with subthreshold levels of *β*‐amyloid in multiple measures of memory (Logical Memory Delayed Recall, *P* = 0.03; Free and Cued Selective Reminding Test, *P* < 0.001), the Preclinical Alzheimer’s Cognitive Composite (*P* = 0.01), and was marginally associated with decreased executive function (Digit Symbol Substitution, *P* = 0.07). Significantly, decreased cognitive scores were associated with suprathreshold levels of *β*‐amyloid, across all measures (*P* < 0.05). The Free and Cued Selective Reminding Test, a list recall memory test, appeared most sensitive to *β*‐amyloid ‐related decreases in average cognitive scores, outperforming all other cognitive domains, including the narrative recall memory test, Logical Memory.

**Interpretation:**

Clinical trials for cognitively unimpaired *β*‐amyloid‐positive individuals will include a large number of individuals where mechanisms downstream from *β*‐amyloid pathology are already activated. These findings have implications for primary and secondary prevention of Alzheimer’s disease.

## Introduction

The preclinical stage of Alzheimer’s disease (AD) is thought to start with the accumulation of *β*‐amyloid (A*β*) pathology and end with the onset of clinical symptoms. The failure of trials directed against symptomatic AD over the last few years has led to an increased interest among researchers and drug developers to study preclinical AD.^1^, ^2^ The hope is that early interventions, initiated before widespread neuronal damage, may be more likely to modify the disease course. To guide these interventions, a detailed understanding of the earliest phases of AD is needed.

In previous studies, preclinical AD has often been defined by abnormal levels of A*β* biomarkers (e.g., using positron emission tomography, PET), typically using thresholds associated with quite advanced A*β* pathology.^3^ We suggest that such overt changes are preceded by subthreshold changes that occur within the normal range of the biomarkers, but which may still have meaningful biological and clinical effects.^4^, ^5^, ^6^, ^7^ A focus on biomarker positivity at conservative thresholds may therefore result in misleadingly late estimates for when preclinical AD starts. In this study, we tested how the principal risk factors for AD, including age and continuous levels of 18F‐florbetapir (a tracer sensitive to fibrillar A*β*) PET, were related to very early changes in cognition, within the normal range.

To further optimize early interventions against AD, we also need to understand the transition from purely asymptomatic preclinical AD into the clinical stage of the disease. Thresholds for cognition that define mild cognitive impairment (MCI) or dementia are too conservative to capture the first subtle changes in cognition. A traditionally identified preclinical AD population (A*β*‐positive cognitively unimpaired) may therefore include a spectrum of participants ranging from no cognitive engagement at all to substantial cognitive dysfunction, albeit less than thresholds for MCI.^8^ The latter “preclinical” AD individuals may already be in a too advanced disease stage to optimally respond to anti‐A*β* treatments (if downstream mechanisms have already been activated, e.g., tau propagation). We therefore tested how 18F‐florbetapir levels were related to subtle cognitive dysfunction in multiple domains in 4432 participants who were screened for participation in the A4 trial.^9^ Specifically, we aimed to evaluate whether (1) there were significant reductions in cognitive scores prior to the threshold for A*β*‐positivity and (2) whether there was a rank order of the decline of specific cognitive domains with respect to continuous levels of 18F‐florbetapir. We hypothesized that A*β* would have subtle effects on cognition, indicating the start of cognitive decline in AD even in a population of participants who were classified as cognitively unimpaired, without MCI or dementia, with subthreshold levels of A*β*.

## Materials and methods

### Participants

Participants who were screened for inclusion in the A4 study^9^, ^10^ (https://clinicaltrials.gov/ct2/show/NCT02008357) were included in this study if they completed an 18F‐florbetapir PET scan, had *APOE* genotype information, completed a battery of neuropsychological testing, scored between 25 and 30 on the Mini‐Mental State Examination (MMSE), had a Clinical Dementia Rating of 0, and were between the ages of 65 and 85. Participants were excluded from the A4 study if they were (1) taking a prescription Alzheimer’s medication (acetylcholinesterase inhibitor and/or memantine); (2) had a current serious or unstable illness, including cardiovascular, hepatic, renal, gastroenterologic, respiratory, endocrinologic, neurologic, psychiatric, immunologic, or hematologic disease or other conditions that could interfere with the study; (3) had a history within the last 5 years of a serious infectious disease affecting the brain (including neurosyphilis, meningitis, or encephalitis) or head trauma resulting in protracted loss of consciousness; (4) had a history within the last 5 years of a primary or recurrent malignant disease, with the exception of resected cutaneous squamous cell carcinoma in situ, basal cell carcinoma, cervical carcinoma in situ, or in situ prostate cancer with normal prostate‐specific antigen posttreatment; (5) had a known history of human immunodeficiency virus (HIV), clinically significant multiple or severe drug allergies, or severe posttreatment hypersensitivity reactions, including but not limited to erythema multiforme major, linear immunoglobulin A dermatosis, toxic epidermal necrolysis, or exfoliative dermatitis; (6) were at serious risk for suicide or had a history within the past 2 years of major depression or bipolar disorder; (7) had a history within the past 5 years of chronic alcohol or drug abuse/dependence; or (8) were residing in a skilled nursing facility or nursing home. Note that participants who did not demonstrate evidence of amyloid in the brain at screening were excluded from the randomized treatment in A4, but participants were included in the current study regardless of their PET scan result.

### 18F‐florbetapir PET imaging

Amyloid PET imaging was done using 18F‐florbetapir data, acquired 50–70 min postinjection. Images were realigned and averaged, and then spatially aligned to a standard space template. 18F‐florbetapir, sampled in a global neocortical region for A*β*, was expressed as an standardized uptake value ratio (SUVR) with a cerebellar reference region.^11^ An A*β*‐negative group was defined as participants with 18F‐florbetapir PET SUVR < 1.10.^12^, ^13^ The quantitative threshold for A*β*+ in our analyses differs from the eligibility for randomization in the A4 study, which used an algorithm involving both SUVR thresholds and visual inspection.

### Cognitive testing

Participants completed a neuropsychological test battery including the Preclinical Alzheimer's Cognitive Composite (PACC),^14^, ^15^ comprising the MMSE, a measure of global cognition, Logical Memory Delayed Recall, a narrative recall memory test, Free and Cued Selective Reminding Test (FCSRT96), a list recall memory test, and the Digit Symbol Substitution Test, a measure of executive function. To calculate the PACC, the individual components were centered on their means and scaled to their standard deviations and summed, calculated using all participants. This sum was then centered on the mean and standard deviation of the sum, calculated using only the A*β*‐negative group. We evaluated the FCSRT96 formulation of the FCSRT as well as the Free Recall portion of the FCRST because of evidence of their sensitivity to early A*β*‐related cognitive changes.^14^, ^16^, ^17^, ^18^

### Statistical analysis

The main outcomes in this study were the PACC and its individual components. The relationship between cognitive scores and 18F‐florbetapir PET, as well as interactions between demographics and 18F‐florbetapir PET, was evaluated. Cognitive scores were modeled using ordinary least squares regression. To evaluate potential nonlinearity in the relationships with cognition, continuous 18F‐florbetapir PET levels were parameterized using monotone cubic splines.^19^ Cubic splines are functions of polynomials allowing flexibility in the estimation of trajectories. Spline models included two knots, with the number of knots selected by the Akaike Information Criterion (AIC).^20^ Statistical significance of the associations between the outcome and predictors was tested using likelihood ratio tests and AIC. A lower value of AIC indicates a better fitting model. Models were adjusted for age, sex, and years of education. Because the range of the MMSE in this cognitively unimpaired population is restricted (25–30), a sensitivity analysis was done assuming a binomial distribution with 30 Bernoulli trials.

**Figure 1:**
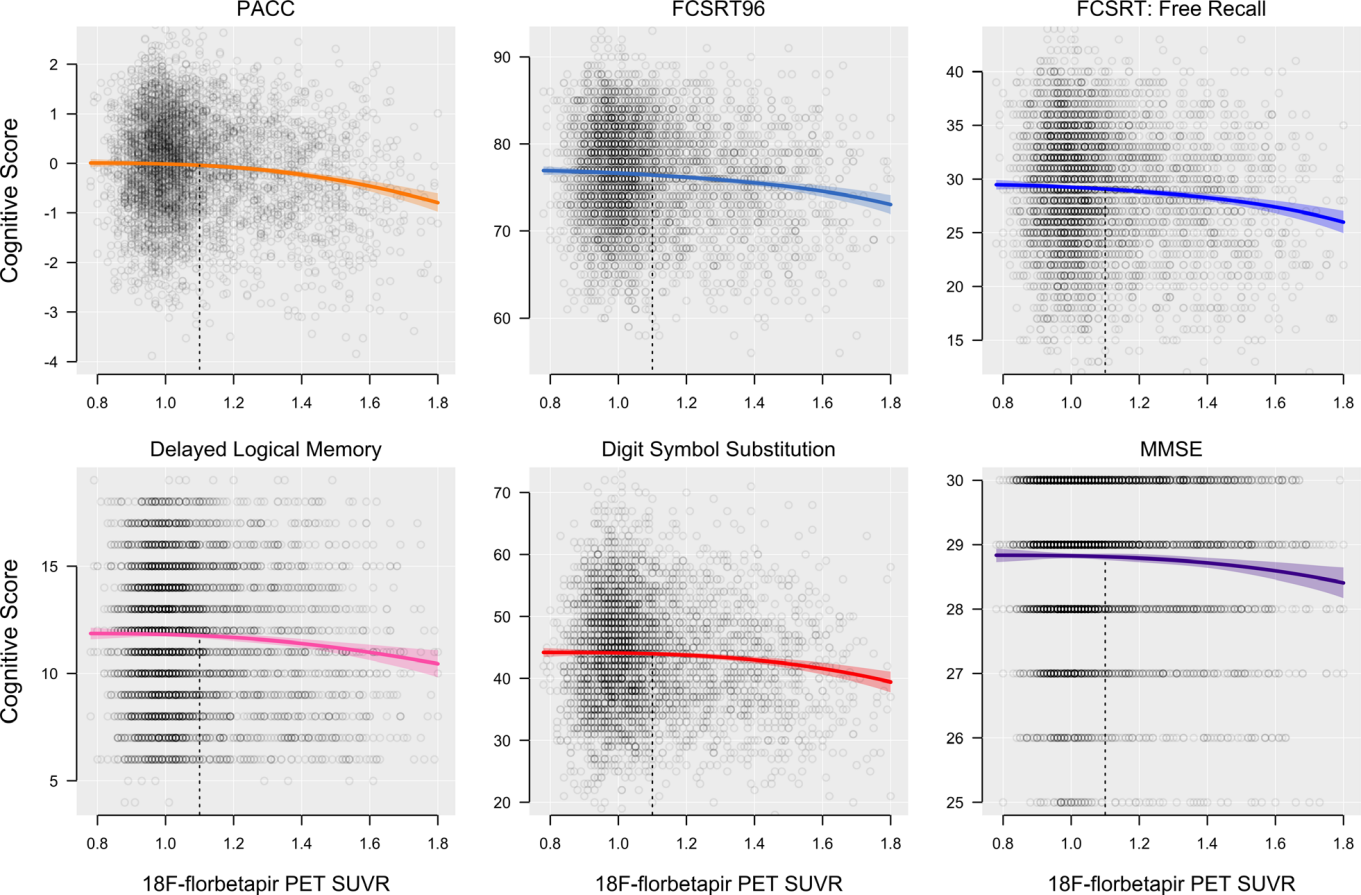
Effects of amyloid PET on cognition. Cognitive scores are plotted against 18F‐florbetapir PET SUVR with mean curves and 95% confidence intervals represented by the shaded region. The vertical dashed line indicates the threshold for A*β*‐positivity (SUVR = 1.10).

We also assessed the measures for their sensitivity to the earliest decreases in cognitive scores. We used a positional variance diagram to depict the ordering of the cognitive measures in terms of decreased score at 18F‐florbetapir SUVR = 1.10 (the threshold for A*β*‐positivity used for these analyses) and also at 18F‐florbetapir SUVR = 1.28 (the median SUVR in A*β*+ participants). The cognitive measures were centered on their estimated means at the lowest 18F‐florbetapir SUVR value and scaled to the standard deviation of the A*β*‐ group. The positional variance diagram shows the proportion of 1000 bootstrap samples in which a particular cognitive measure appears in a particular position in the central ordering, ranging from 0 (white or no shading, in Fig. 3) to 1 (blue shading). Cognitive measures are ordered by their most frequently estimated position with uncertainty captured by the transparency of the shading.

Additionally, permutation tests were performed to estimate the statistical significance of the magnitude of the decrease in cognitive scores at 18F‐florbetapir SUVR = 1.10 and separately at SUVR = 1.28, compared with the scores at the lowest level of 18F‐florbetapir, SUVR = 0.78. Spline model‐estimated decreases in cognitive scores from SUVR = 0.78 to SUVR = 1.10 (and separately, to SUVR = 1.28) based on permuted values of 18F‐florbetapir in each bootstrap sample were used to obtain a null distribution of cognitive changes. A null distribution results because the 18F‐florbetapir values are attributed at random via permutation. *P*‐values were then calculated as the proportion of cognitive changes from the null distribution that were equal or greater than the observed changes estimated using the true 18F‐florbetapir values.

Associations between demographics and 18F‐florbetapir PET SUVR were assessed using Spearman correlation for continuous variables and a Kruskal‐Wallis test for categorical variables. Changes in AIC (*Δ*AIC) < −2 were considered a meaningful^21^ improvement with respect to the more parsimonious model and *P*‐values < 0.05 were considered significant. All analyses were done in R v3.6.0 (http://www.r-project.org); splines were estimated using the splines2 package.

### Standard protocol approvals, registrations, and patient consents

This study was approved by the Institutional Review Boards of all of the participating institutions. Informed written consent was obtained from all participants at each site.

## Results

### Cohort characteristics

A total of 4432 cognitively unimpaired adults were included in the study. They were 71.3 years old on average (interquartile range: 67.5–74.2), 59.4% female, had an average of 16.6 years of education (interquartile range: 15 to 18), and 34.1% were A*β*+. 18F‐florbetapir SUVR was significantly correlated with increasing age (*ρ* = 0.08, *P* < 0.001) and *APOE* *ε*4 status (mean SUVR in *ε*4‐ = 1.05, mean SUVR in *ε*4+ = 1.18, *P* < 0.001), whereas only marginally associated with sex (mean SUVR in males = 1.09, mean SUVR in females = 1.10, *P* = 0.06). 18F‐florbetapir SUVR was not associated with years of education (*ρ* = −0.009, *P* = 0.56).

### Cognition

**Figure 2:**
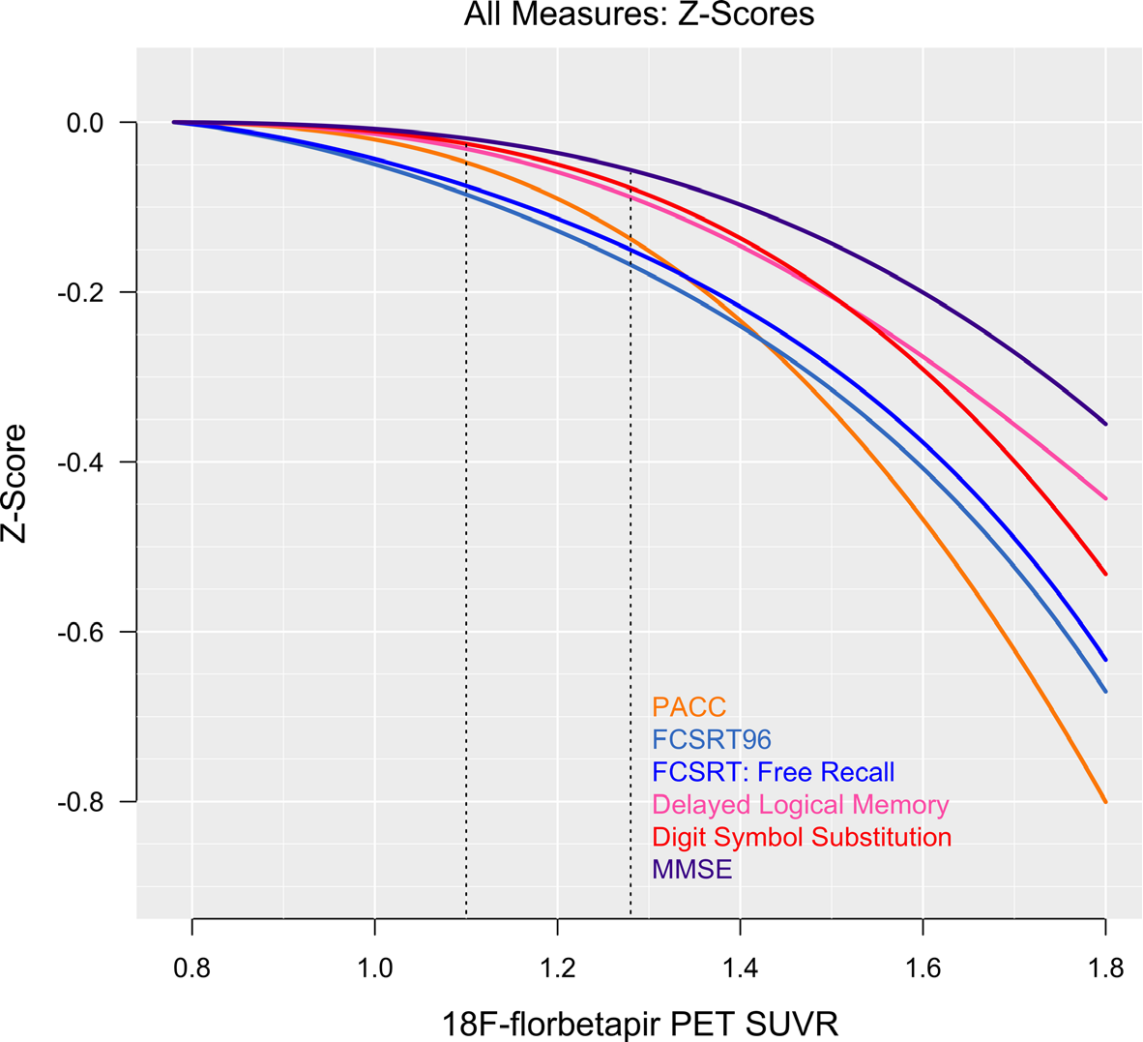
All cognitive measures. The summary panel shows the *z*‐scores of all measures. The vertical lines indicate the threshold for A*β*‐positivity and the median 18F‐florbetapir PET SUVR of the A*β* + group (1.28).

The PACC and each individual cognitive component were significantly associated with 18F‐florbetapir SUVR (*P* < 0.01, *Δ*AIC < −8). Each cognitive domain trajectory is shown in Figures 1 and 2. In Table 1, mean cognitive scores at the threshold for A*β*+ (18F‐florbetapir SUVR = 1.10) and at the median of the A*β*+ participants (18F‐florbetapir SUVR = 1.28) are summarized, as well as the decreases in cognitive scores compared to the values at the floor of 18F‐florbetapir PET (18F‐florbetapir SUVR = 0.78). The results from the sensitivity analysis for MMSE assuming a binomial distribution were the same as the main analysis, with mean estimates and 95% confidence intervals almost perfectly overlapping.

**Table 1:** Cognitive scores, standard deviations, and effect sizes.

	Mean (SUVR = 0.78)	SD	Mean (SUVR = 1.10)	*Δ*_1.10_ *	*P*	Effect size (*Δ*_1.10_/SD)	Mean (SUVR = 1.28)	*Δ*_1.28_	*P*	Effect size (*Δ*_1.28_/SD)
PACC	0.008	1.00	−0.04	−0.05	0.01	−0.05	−0.13	−0.14	0.001	−0.14
FCSRT96	76.93	5.81	76.44	−0.49	<0.001	−0.09	75.94	−0.99	<0.001	−0.17
FCSRT (Free Recall)	29.47	5.50	29.06	−0.41	<0.001	−0.08	28.63	−0.84	<0.001	−0.15
Delayed logical memory	11.86	3.17	11.76	−0.10	0.03	−0.03	11.58	−0.29	0.003	−0.09
Digit symbol substitution	44.20	8.96	43.98	−0.23	0.07	−0.03	43.49	−0.71	0.01	−0.08
MMSE	28.83	1.20	28.81	−0.02	0.11	−0.02	28.77	−0.07	0.05	−0.06

* Refers to the change in cognitive score from SUVR 0.78 to SUVR 1.10.

Delayed Logical Memory, FCSRT96, FCSRT Free Recall, and the PACC all showed significant subthreshold decreases from SUVR 0.78 to SUVR 1.10 (*P* < 0.03, Table 1), and Digit Symbol Substitution showed a marginally significant decrease (*P* = 0.07). All cognitive measures decreased significantly from SUVR 0.78 to SUVR 1.28 (*P* < 0.05).

**Figure 3:**
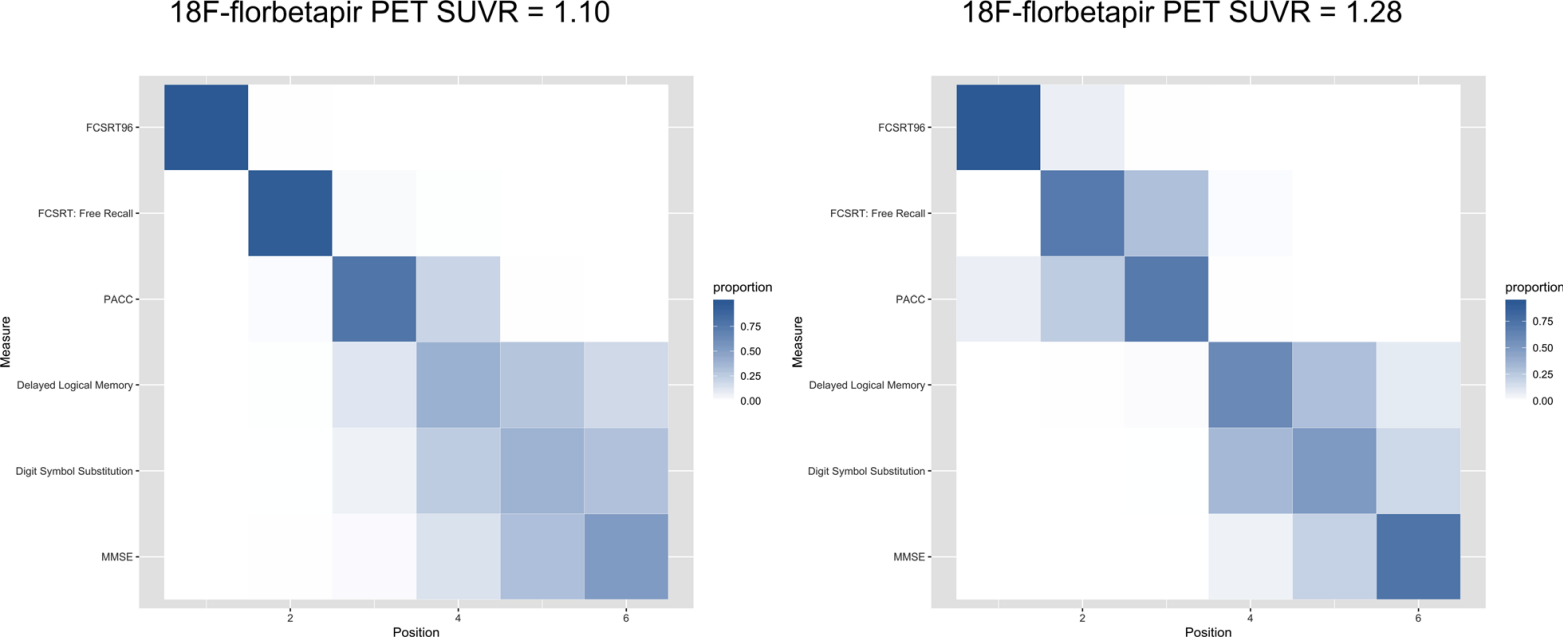
Order of cognitive measures by 18F‐florbetapir PET SUVR. Positional variance diagrams to depict the ordering of the cognitive measures in terms of decreased score at 18F‐florbetapir SUVR = 1.10 and 1.28. The cognitive measures were centered on their estimated means at the lowest 18F‐florbetapir SUVR value and scaled to the standard deviation of the A*β*‐ group. The positional variance diagram shows the proportion of 1000 bootstrap samples in which a particular cognitive measure appears in a particular position in the central ordering, ranging from 0 (white or no shading) to 1 (blue shading). Cognitive measures are ordered by their most frequently estimated position with uncertainty captured by the transparency of the shading.

Positional variance diagrams, depicting the order of all cognitive measures in terms of decrease in scores with increasing SUVR, are shown in Figure 3. At the threshold for A*β*+, FCSRT96 consistently (99% of the time) showed the greatest decrease in cognitive score, followed by the FCSRT Free Recall and the PACC (also seen in Figs. 1 and 2). At the median 18F‐florbetapir uptake in A*β*+ (SUVR 1.28), the order remained the same. MMSE was consistently in the fifth position.

### A*β* demographic interactions to predict the PACC

**Figure 4:**
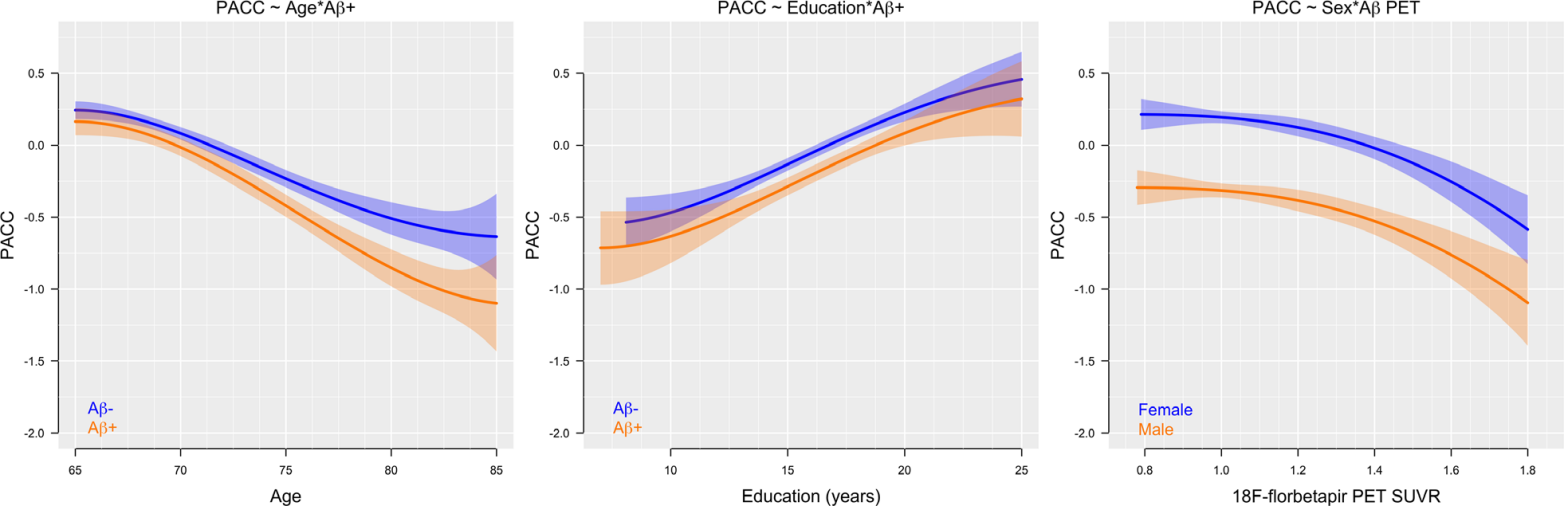
Interactions between A*β* PET and demographic factors predicting PACC scores. Adjusting for sex and education, there was a significant interaction between A*β*‐positivity and age to predict decreasing PACC scores (*P* = 0.02, *Δ*AIC = −3.8, left panel). The association between A*β*‐positivity and decreasing PACC scores did not differ by years of education (*P* = 0.99, *Δ*AIC = 6.0, middle panel) or sex after adjusting for age and education (*P* = 0.95, *Δ*AIC = 5.6, right panel).

There was a significant interaction between 18F‐florbetapir and age to predict decreasing PACC scores, where steeper decline with age was observed in the A*β*+ participants (*P* = 0.02, *Δ*AIC = −3.8). The association between 18F‐florbetapir and decreasing PACC scores did not differ by years of education (*P* = 0.99, *Δ*AIC = 6.0) or sex (*P* = 0.95, *Δ*AIC = 5.6), although for a given level of 18F‐florbetapir, females had higher scores compared with males. All interactions are shown in Figure 4.

## Discussion

The main finding of this study was that subtle decline in cognition had already occurred as a function of subthreshold levels of A*β* in cognitively unimpaired individuals. Among included tests, average scores on the FCSRT96 decreased first, with respect to A*β* accumulation. This suggests that the effects of A*β* on cognition start early, in specific memory tests, with over half of individuals in a traditionally recruited “preclinical AD” population being at risk for subtle cognitive effects already at baseline. If the goal is to interfere very early in the disease process before downstream pathways are activated, primary or secondary AD prevention trials may therefore both (1) aim at younger people (below age 65–70 years) and (2) exclude individuals with high levels of A*β* accumulation.

The effect of A*β* on cognition in this cognitively unimpaired population is in line with previous large‐scale studies. For example, in a recent meta‐analysis of three large studies (not including A4), we found that A*β*‐positive cognitively unimpaired individuals had significantly lower scores on several cognitive tests compared to A*β*‐negative individuals already at baseline.^22^ This is no surprise, both because there are no clear boundaries between the clinical stages of AD,^23^ and because one may expect performance on cognitive tests to change gradually within the normal range due to AD before reaching the threshold for an MCI diagnosis, indicating cognitive impairment. Although cognitive changes in the subthreshold range of A*β* are quite small, and not clinically meaningful in terms of effect size, they mark the initial descent of cognitive decline. This large sample enables the observation of the gradual and continuous decrease in cognitive scores with increasing A*β*, leading to the more substantial decrease in cognitive scores at the median level of the A*β*‐positive group and beyond. It is also possible that these initial signs of cognitive dysfunction are due primarily to synaptic dysfunction caused by A*β* toxicity, prior to overt neuronal degeneration.^24^ However, if the decline in cognition is driven not by A*β* accumulation, but rather by changes in tau and neurodegeneration,^25^ the earliest changes in cognition may indicate the point in the disease when mechanisms downstream to A*β* have become activated. This may also be a point when anti‐A*β* treatments are less likely to successfully modify the disease course. This may pose a significant problem for ongoing and planned anti‐A*β* trials focused on preclinical AD, since they may include significant numbers of participants with too advanced disease to respond to this class of treatments.

The FCSRT96, included in the PACC for its sensitivity to early memory changes,^14^, ^16^, ^17^, ^18^ appeared more sensitive to A*β*‐associated cognitive decline compared with the other components of the PACC. Notably, the FCSRT96, a list recall memory test, outperformed the narrative recall memory test, Delayed Logical Memory (Figs. 1, 2, 3). The effect size of the FCSRT96 of the reduction in average cognitive score between the lowest 18F‐florbetapir SUVR levels and the 1.10 threshold for A*β*‐positivity was −0.09 (Table 1), more than double the effect size of any of the other three components of the PACC, including Delayed Logical Memory. Because the PACC is an average of the four cognitive tests, the early decline in PACC scores, with respect to 18F‐florbetapir SUVR, appeared largely driven by the early changes in the FCSRT96. It was not until the trajectories of the remaining three cognitive tests showed an accelerated descent with respect to 18F‐florbetapir SUVR that the PACC converged and eventually overtook the FCSRT96 near A*β* SUVR = 1.4 (Fig. 2).

There was an age‐dependent effect of A*β* on PACC scores, with increased separation between A*β*‐ and A*β*+ groups with older age. This is likely due to older age representing a longer time spent with a significant A*β* burden, or due to the presence of comorbidities in older age which may lower the threshold for when A*β* pathology leads to cognitive decline. All participants in this study have remained cognitively unimpaired to at least 65 years of age, suggesting that none of them are on an early AD path where A*β*‐positivity at a younger age might be expected to be associated with more aggressive disease and more rapid progression.^26^ In contrast, A*β*‐related reduction of PACC scores did not differ by sex. This is consistent with some, but not all, reports of sex‐related associations with A*β* and cognition.^22^, ^27^ While females generally outperform males in most cognitive domains,^28^ in the current setting, the decrease in PACC scores with increasing levels of A*β* occurred in parallel with males and females. In a longitudinal setting where participants progress beyond the preclinical stage, tau accumulation, neurodegeneration, and rates of cognitive decline may become sex‐dependent, resulting in the sex differences observed in other cohorts.^27^, ^29^

This study has several limitations. Inclusion criteria restricted participation to those over 65 years of age, and a restricted performance range on cognitive testing, limiting analyses to the effect of emerging A*β* pathology within late‐onset preclinical AD. Although the study sample size was large, without tau PET or measures of cerebrovascular disease, we were limited to only part of the picture of cognitive changes in preclinical AD. Importantly, without longitudinal information, these results do not apply to individual trajectories of amyloid deposition or changes in cognition. Longitudinal follow‐up of middle‐aged individuals, with low and intermediate levels of amyloid, will be required to further clarify the earliest cognitive changes associated with A*β* accumulation. Finally, participation in the A4 study may represent a unique cohort that may reduce the generalizability of the results outside of clinical trials.

With a large sample size, subtle estimates of cognitive dysfunction can be estimated with high precision, as is required to detect the small cognitive changes associated with subthreshold increases of A*β* pathology. These findings suggest that subthreshold changes that occur within the normal range of A*β* have meaningful biological and clinical implications for defining preclinical AD. An alternative threshold for A*β*‐positivity, prior to the onset of subtle cognitive dysfunction, may be required to identify individuals in the earliest stages of the disease, for whom early treatment may be the most beneficial.

## Author Contributions

PSI contributed to the conception and design of the study, analysis of the data, and drafting of the manuscript and the figures. NM contributed to the conception and design of the study and drafting of the manuscript. RS and MCD contributed to the acquisition of the data and drafting of the manuscript. OH contributed to the drafting of the manuscript.

## Conflict of Interest

Mr Insel, Mattsson, and Hansson report no conflict of interest. Donohue reports grants from NIH, grants from Eli Lilly and Co, grants from Alzheimer's Association, grants from Accelerating Medicines Partnership, from GHR Foundation, nonfinancial support from Avid, nonfinancial support from Cogstate, during the conduct of the study; personal fees from Biogen, grants and other from Janssen, personal fees from Neurotrack, personal fees from Roche, outside the submitted work. Sperling reports personal fees from AC Immune, personal fees from Biogen, personal fees from Janssen, personal fees from Neurocentria, grants from Eli Lilly, grants from Janssen, grants from NIA, grants from Alzheimer’s Association, personal fees and other from Novartis, personal fees and other from AC Immune, outside the submitted work.
